# Impact of the COVID-19 Pandemic on ST-Elevation Myocardial Infarction Management in Hunan Province, China: A Multi-Center Observational Study

**DOI:** 10.3389/fcvm.2022.851214

**Published:** 2022-03-31

**Authors:** Liang Tang, Zhao-jun Wang, Xin-qun Hu, Zhen-fei Fang, Zhao-fen Zheng, Jian-ping Zeng, Lu-ping Jiang, Fan Ouyang, Chang-hui Liu, Gao-feng Zeng, Yong-hong Guo, Sheng-hua Zhou

**Affiliations:** ^1^Department of Cardiology, The Second Xiangya Hospital of Central South University, Changsha, China; ^2^Hunan Provincial People’s Hospital, The First Affiliated Hospital of Hunan Normal University, Changsha, China; ^3^Xiangtan Central Hospital, Xiangtan, China; ^4^Changsha Central Hospital, Changsha, China; ^5^Zhuzhou Central Hospital, Zhuzhou, China; ^6^The First Affiliated Hospital of University of South China, Hengyang, China; ^7^The Second Affiliated Hospital of University of South China, Hengyang, China; ^8^Department of Geriatric, The Second Xiangya Hospital of Central South University, Changsha, China

**Keywords:** COVID-19, ST-segment elevation myocardial infarction, primary percutaneous coronary intervention, thrombolysis, outcomes

## Abstract

**Background:**

This study aimed to investigate the impact of the COVID-19 pandemic on ST-segment elevation myocardial infarction (STEMI) care in China.

**Methods:**

We conducted a multicenter, retrospective cohort study in Hunan province (adjacent to the epidemic center), China. Consecutive patients presenting with STEMI within 12 h of symptom onset and receiving primary percutaneous coronary intervention, pharmaco-invasive strategy and only thrombolytic treatment, were enrolled from January 23, 2020 to April 8, 2020 (COVID-19 era group). The same data were also collected for the equivalent period of 2019 (pre-COVID-19 era group).

**Results:**

A total of 610 patients with STEMI (COVID-19 era group *n* = 286, pre-COVID-19 era group *n* = 324) were included. There was a decline in the number of STEMI admissions by 10.5% and STEMI-related PCI procedures by 12.7% in 2020 compared with the equivalent period of 2019. The key time intervals including time from symptom onset to first medical contact, symptom onset to door, door-to-balloon, symptom onset to balloon and symptom onset to thrombolysis showed no significant difference between these two groups. There were no significant differences for in-hospital death and major adverse cardiovascular events between these two groups.

**Conclusion:**

During the COVID-19 pandemic outbreak in China, we observed a decline in the number of STEMI admissions and STEMI-related PCI procedures. However, the key quality indicators of STEMI care were not significantly affected. Restructuring health services during the COVID-19 pandemic has not significantly adversely influenced the in-hospital outcomes.

## Introduction

In late December 2019, an outbreak of coronavirus disease 2019 (COVID-19) caused by severe acute respiratory syndrome coronavirus 2 (SARS-CoV-2) occurred in Wuhan, China (1, 2). Within 3 months since the outbreak, COVID-19 has emerged as a pandemic and an international public health crisis (3). According to the dynamic real-time information provided by Johns Hopkins University Coronavirus Resource Center, as of January 7, 2022, the pandemic has infected over 303,204,268 people and caused 5,479,893 deaths globally (4). The ongoing pandemic of COVID-19 has imposed a serious threat on public health and the economy worldwide.

ST-segment elevation myocardial infarction (STEMI) remains a leading cause of death worldwide (5). Improvement in clinical outcomes after STEMI depends greatly on the timely effective reperfusion therapy. Primary percutaneous coronary intervention (PPCI) is the preferred reperfusion strategy and is the current standard of care for STEMI (6). However, the COVID-19 pandemic inevitably poses a severe challenge to the emergent care of STEMI patients, as the regional STEMI-network was reorganized to assist COVID-19 patients, and the screening and infectious control of COVID-19 procedures required to prevent nosocomial infection may substantially defer PPCI (7–9). Recently, the American College of Cardiology’s Interventional Council and Society of Cardiovascular Angiography and Intervention have issued a statement on the management of STEMI in the context of the COVID-19 pandemic and it continues to recommend PPCI as the standard treatment of STEMI patients with unconfirmed COVID-19 status (10). In contrast, the Chinese Society of Cardiology has issued a consensus on the management of STEMI during the COVID-19 pandemic and recommended a strategy of thrombolytic therapy over PPCI due to concerns of resource allocation, as well as challenges in transfer of patients to facilities that perform PPCI (11).

To date, while there are isolated local and regional level reports that the COVID-19 pandemic is associated with a reduction in both presentations with STEMI and PPCI procedures (7, 8), there have been limited data regarding its impact on real-world reperfusion strategies decision making, key indicators of STEMI care, and clinical outcomes. Therefore, the present investigation was undertaken to investigate the real-world impact of the COVID-19 pandemic on time-sensitive STEMI care delivery in Hunan province, China, a so-called “hot-spot” province (adjacent to the epidemic center Wuhan) where the impact would be expected to be most pronounced and lab results.

## Materials and Methods

### Study Design and Population

We conducted a multicenter, retrospective study involving 13 tertiary care cardiac catheterization centers in Hunan province, China. Consecutive patients, presenting with STEMI within 12 h of symptom onset and receiving reperfusion therapy with PPCI, pharmaco-invasive strategy, and only thrombolytic treatment, were enrolled from January 23, 2020 to April 8, 2020, when the city of Wuhan was on lockdown to constrain the spread of the virus. A group of STEMI patients from the equivalent period of last year (i.e., January 23, 2019 to April 8, 2019; pre-COVID-19 era group) was used as control.

During the COVID-19 pandemic, all STEMI patients were screened for COVID-19 first. All admitted patients were required to undergo temperature checks and complete an epidemiological survey at prescreening triage station, which was set up at the entrance of the emergency department. For patients with suspected COVID-19 infection, rapid chest scans and routine blood tests were performed. A nasopharyngeal swab was performed if the condition of the patient allows it. Patients were transferred to a COVID-19-designated hospital if COVID-19 is confirmed. In this study, patients with confirmed or suspected COVID-19 were excluded. Besides, Patients were excluded from the analysis if they presented with ischemic time > 12 h or unknown time, combined with neoplastic disease, discharged to other medical facilities within 48 h, received no acute reperfusion, or had records with missing or incomplete data. STEMI patients were classified into two groups: COVID-19 era group and pre-COVID-19 era group according to the time admitted in hospitals. Patients who underwent PCI were further categorized according to whether they received PPCI or pharmaco-invasive strategy to analyze the procedural characteristics and key time indicators. The diagnosis of STEMI was made based on the fourth universal definition (12). The pharmaco-invasive strategy was defined as fibrinolysis combined with routine early PCI strategy (in case of successful fibrinolysis) or rescue PCI (in case of failed fibrinolysis) (6). The study was conducted in accordance with the Declaration of Helsinki and approved by the local hospital Institutional Review Board, and the need for informed consent for using the medical records was waived owing to the retrospective nature of the study.

### Data Collection

The clinical data were collected by trained staff reviewing the medical records of all patients. Data were collected retrospectively, in an anonymized fashion without any sensitive data. We collected detailed baseline variables including demographics, cardiovascular risk factors, medical history, physical findings, and Killip classification on admission, early medical treatments (within 24 h after hospital arrival), and laboratory tests. Treatment timelines delay including symptom onset to first medical contact (FMC), symptom onset to door, door to balloon, symptom onset to balloon, and symptom onset to thrombolysis time. In addition, angiographic and procedural characteristics were assessed.

### Clinical Outcomes

All adverse clinical events were adjudicated through the use of original source documentation by an independent committee that was unaware of the treatment allocation. The primary outcome of interest was the number of STEMI admissions and STEMI-related PCI (including PPCI, rescue PCI, and routine early PCI) procedures during Wuhan lockdown and the equivalent period in 2019. The secondary outcomes were in-hospital all-cause mortality and major adverse cardiovascular events (MACEs), which were defined as a composite of death, non-fatal reinfarction, target vessel revascularization, new-onset congestive heart failure, and stroke during hospitalization (13, 14).

### Statistical Analysis

Continuous data were reported as median with 25th and 75th percentiles (interquartile range, IQR) and compared by the Mann–Whitney *U* test. Categorical data were expressed as numbers and percentages and compared by the chi-square test or Fisher’s exact test. Multivariate logistic regression analysis was used to identify independent predictors of in-hospital mortality and MACEs. All statistical tests were performed using SPSS software, version 24.0 (SPSS Inc., Chicago, IL, United States). A *P* value of <0.05 was regarded as statistically significant.

## Results

### Baseline Characteristics

**FIGURE 1:**
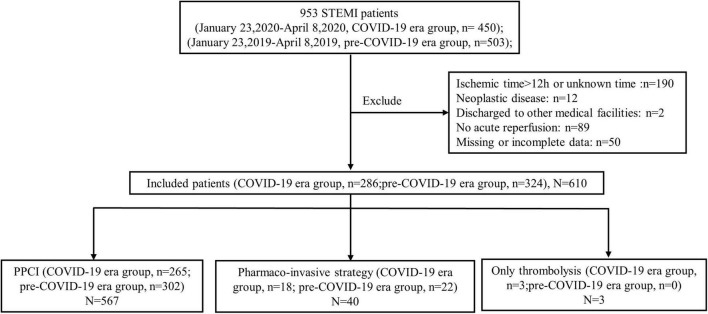
Study flow chart. STEMI, ST-segment elevation myocardial infarction; COVID-19, coronavirus disease 2019; PPCI, primary percutaneous coronary intervention.

**FIGURE 2:**
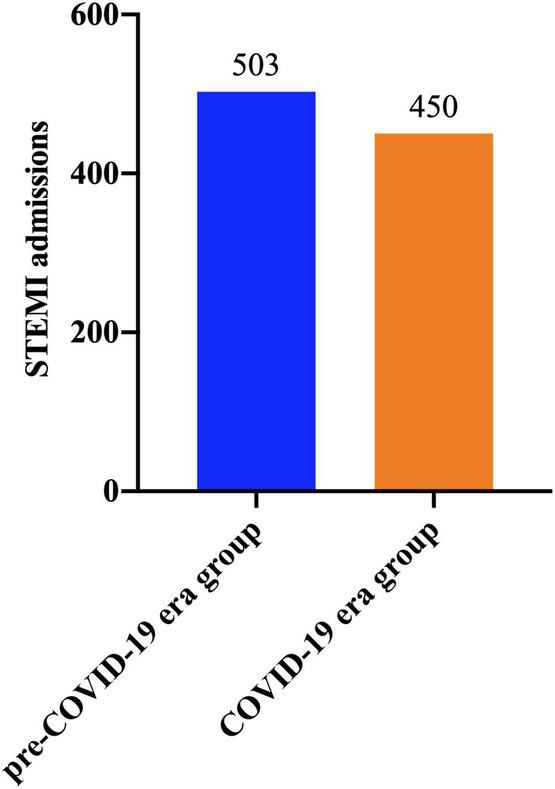
Admissions for STEMI during Wuhan lockdown and equivalent time period in 2019. STEMI, ST-segment elevation myocardial infarction; COVID-19, coronavirus disease 2019.

**FIGURE 3:**
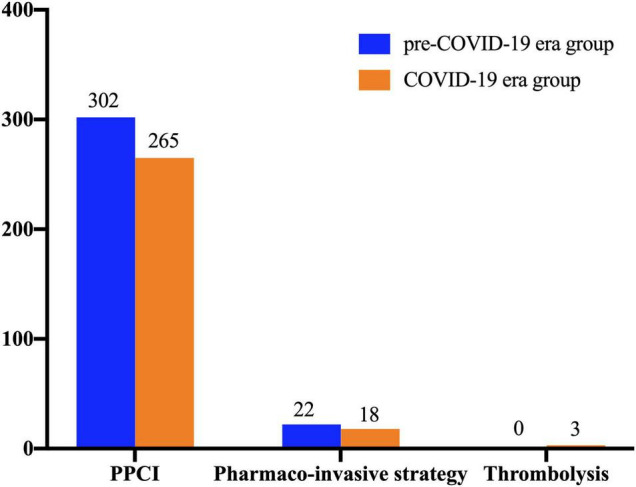
Reperfusion strategy for STEMI patients during Wuhan lockdown and equivalent time period in 2019. STEMI, ST-segment elevation myocardial infarction; COVID-19, coronavirus disease 2019; PPCI, primary percutaneous coronary intervention.

A total of 953 consecutive patients (COVID-19 era group *n* = 450, pre-COVID-19 era group *n* = 503) were admitted for STEMI during the described time frames. Of these, no patient was confirmed or suspected COVID-19 and 610 patients (COVID-19 era group *n* = 286, pre-COVID-19 era group *n* = 324) fit the inclusion criteria for this study (Figure 1). Among this study population, 567 (93.0%) patients received PPCI. Forty (6.6%) patients received pharmaco-invasive treatment including routine early PCI (*n* = 18) and rescue PCI (*n* = 22). When comparing the study period of 2020 to the equivalent period of 2019, a reduction of 10.5% in STEMI admissions was observed (Figure 2). Also, there was a 12.7% decline in the number of all STEMI-related PCI procedures (including PPCI, rescue PCI and routine early PCI) when compared to the same time interval in 2019 (Figure 3). The remaining 3 (0.5%) patients received only thrombolysis treatment.

Demographic, baseline clinical characteristics and laboratory variables of the enrolled patients are listed in Table 1. Compared with the pre-COVID-19 era group, the COVID-19 era group was more likely to have a history of previous PCI. Otherwise, there were no significant differences between the two groups in terms of patient demographics, medical history, the prevalence of coronary risk factors, physical findings on admission and concomitant medications. Patients were presented with a slightly higher cardiac troponin I (cTnI) level on admission during the COVID-19 pandemic era compared with the pre-COVID-19 era. Patients in the COVID-19 era group tended to have a lower blood urea nitrogen, low-density lipoprotein cholesterol and C-reactive protein level on admission (Table 1).

**TABLE 1:** Baseline clinical characteristics.

	COVID-19 group (*n* = 286)	Pre-COVID-19 group (*n* = 324)	Statistic	*P* value
**Demographics**				
Age (years)	63 (53.5–70)	63 (53–75)	−0.727^‡^	0.467
Male sex, *n* (%)	229 (80.1)	250 (77.2)	0.763^†^	0.383
**Cardiovascular risk factors, *n* (%)**				
Diabetes mellitus	51 (17.8)	77 (23.8)	3.225^†^	0.073
Hypertension (>140/90 mmHg)	143 (50.0)	148 (45.7)	1.137^†^	0.286
Hyperlipidemia	90 (31.5)	113 (34.9)	0.795^†^	0.373
Current smoker	129 (45.1)	164 (50.6)	1.849^†^	0.174
**Number of cardiovascular risk factors**			0.658^†^	0.883
≥3	48 (16.8)	60 (18.5)		
2	99 (34.6)	106 (32.7)		
1	90 (31.5)	98 (30.2)		
0	49 (17.1)	60 (18.5)		
**Medical history, *n* (%)**				
History of PCI	16 (5.6)	6 (1.9)	6.120^†^	0.013
History of CABG	0 (0.0)	0 (0.0)	—	—
Previous MI	8 (2.8)	10 (3.1)	0.440^†^	0.833
**Physical findings on admission**				
Systolic blood pressure (mm Hg)	126 (110–145.25)	127 (110–141.5)	−0.126^‡^	0.900
Heart rate (beats/min)	77.96 ± 18.083	78.43 ± 17.135	−0.329*	0.742
**Killip classification on admission, *n* (%)**			2.836^†^	0.242
Class I	191 (66.8)	225 (69.4)		
Class II	71 (24.8)	64 (19.8)		
Class III–IV	24 (8.4)	35 (10.8)		
**Medication within 24 h of hospital arrival, *n* (%)**				
Aspirin	285 (99.7)	322 (99.4)	−	1.000
P2Y12 receptor inhibitor	286 (100.0)	323 (99.7)	−	1.000
GP IIb/IIIa receptor inhibitor	149 (52.1)	151 (46.6)	1.834^†^	0.176
β-blockers	230 (80.4)	279 (86.1)	3.562^†^	0.059
Statins	271 (94.8)	314 (96.9)	1.800^†^	0.180
Angiotensin-converting enzyme inhibitors/Angiotensin receptor blockers	211 (73.8)	240 (74.1)	0.007^†^	0.933
**Laboratory tests**				
White blood cell × 10^9^/L	9.8 (8.0–12.9)	10.6 (8.0–13.3)	−0.560^‡^	0.575
Neutrophil × 10^9^/L	7.9 (5.7–10.9)	8.4 (5.8–10.7)	−0.402^‡^	0.608
Lymphocyte × 10^9^/L	1.3 (1.0–1.8)	1.3 (0.9–2.0)	−0.553^‡^	0.580
Platelets × 10^9^/L	204.0 (169.0–250.5)	209.5 (177.0–243.3)	−0.097^‡^	0.923
Hs-cTnI (pg/ml)	5.3 (0.8–23.8)	3.0 (0.3–18.2)	−2.707^‡^	0.007
CK (U/L)	877.0 (225.5–2590.7)	939.1 (236.0–2199.0)	−0.243^‡^	0.808
CK-MB (U/L)	92.7 (27.2–232.0)	99.0 (34.6–235.7)	−0.704^‡^	0.482
BNP (pg/ml)	100.0 (31.3–225.1)	105.0 (67.3–419.3)	−1.687^‡^	0.092
NT-proBNP (pg/ml)	560.4 (160.3–1727.5)	392.0 (91.4–1541.0)	−1.534^‡^	0.125
ALT (U/L)	35.2 (24.1–60.0)	39.8 (26.0–61.4)	−1.144^‡^	0.253
AST (U/L)	91.5 (37.9–208.6)	114.5 (38.5–247.9)	−1.378^‡^	0.168
BUN (mmol/L)	5.7 (4.6–7.2)	6.4 (5.0–8.0)	−3.181^‡^	0.001
TC (mmol/L)	4.7 (3.9–5.5)	4.7 (4.0–5.4)	−0.647^‡^	0.517
TG (mmol/L)	1.5 (1.0–2.3)	1.7 (1.1–2.4)	−1.224^‡^	0.221
HDL-C (mmol/L)	1.0 (0.9–1.2)	1.1 (0.9–1.3)	−1.735^‡^	0.083
LDL-C (mmol/L)	2.8 (2.2–3.5)	3.1 (2.5–3.7)	−2.557^‡^	0.011
Hs-CRP (mg/L)	4.2 (1.7–11.7)	7.6 (2.9–21.4)	−2.737^‡^	0.006
PT (s)	12.5 (11.1–14.6)	12.0 (10.3–15.3)	−2.539^‡^	0.011
APTT (s)	34.1 (27.5–55.5)	32.4 (26.8–41.1)	−2.292^‡^	0.022
D-dimer (mg/L)	0.4 (0.2–1.9)	0.4 (0.2–0.8)	−1.830^‡^	0.067

Data are expressed as mean ± SD, as percentages, or as median (Q_1_, Q_3_). *t value; ^†^χ^2^ value; ^‡^Z value. —: Data not available (Fisher exact test). COVID-19, coronavirus disease 2019; PCI, percutaneous coronary intervention; CABG, coronary artery bypass graft; MI, myocardial infarction; Hs-cTnI, high sensitivity cardiac troponin I; CK, creatine phosphokinase; CK-MB, creatine phosphokinase-MB; BNP, B-type natriuretic peptide; NT-proBNP, N terminal pro-hormone BNP; ALT, alanine transaminase; AST, aspartate transaminase; BUN, blood urea nitrogen; TC, total cholesterol; TG, triglycerides; HDL-C, high-density lipoprotein cholesterol; LDL-C, low-density lipoprotein cholesterol; Hs-CRP, high-sensitivity C-reactive protein; PT, prothrombin time; APTT, activated partial thromboplastin time.

The baseline angiographic features and procedural data are summarized in Table 2. For patients who received PPCI, the time from symptom onset to FMC, symptom onset to door and symptom onset to balloon were not substantially longer during the COVID-19 pandemic era. The door-to-balloon time and the total procedure time were similar between two groups. For pharmaco-invasive patients, the key time interval, including the time from symptom onset to FMC, symptom onset to door and symptom onset to thrombolysis, showed no significant increase during the COVID-19 pandemic era. Patients who received PPCI were less likely to have a right coronary artery occlusion, multivessel disease, and radial access during the COVID-19 pandemic era compared with the pre-COVID-19 era. Moreover, patients who received PPCI during the COVID-19 pandemic had a greater proportion of direct stenting and thrombus aspiration. Among patients admitted with PPCI, the intra-aortic balloon pump (IABP) use and extracorporeal membrane oxygenation use were no difference during the COVID-19 pandemic and pre-COVID-19 era. A high procedural success rate (97.7 vs. 99.0%) and low complications rate (1.5 vs. 1.3%) were similarly observed between two groups. Among patients who received pharmaco-invasive strategy, no significant difference was observed between the two groups with regard to the location of culprit artery, initial and final TIMI flow grade, and prevalence of multivessel diseases. IABP was less used in these patients during the COVID-19 pandemic era.

**TABLE 2:** Angiographic characteristics and procedural data.

	PPCI (*n* = 567)				Pharmaco-invasive (*n* = 40)			
	COVID-19 era group (*n* = 265)	Pre-COVID-19 era group (*n* = 302)	Statistic	*P* value	COVID-19 era group (*n* = 18)	Pre-COVID-19 era group (*n* = 22)	Statistic	*P* value
**Time delays, min**								
Symptom onset to FMC	141 (67–282)	147 (70.25–270)	−0.292^‡^	0.77	88 (30–121.75)	72 (30–193.75)	−0.015^‡^	0.988
Symptom onset to door	185 (105.75–301)	210 (103–327.5)	−0.668^‡^	0.504	349 (220–592.5)	382.5 (186.25–579)	−0.184^‡^	0.854
Door-to-balloon	79 (61–102.5)	77 (55.5–99.5)	−0.855^‡^	0.393	−	−	−	−
Symptom onset to balloon	274 (177.75–372.75)	278.5 (182.75–425.75)	−0.957^‡^	0.339	−	−	−	−
Total procedure time	55 (43–72)	53 (42–67)	−1.083^‡^	0.279	−	−	−	−
symptom onset to thrombolysis	−	−	−	−	139 (70.25–185.5)	120 (70–225.5)	−0.155^‡^	0.877
**Infarct-related artery, *n* (%)**								
LM	18 (6.8)	14 (4.6)	1.233^†^	0.267	1 (5.6)	0 (0.0)	−	0.45
LAD	150 (56.6)	170 (56.3)	0.006^†^	0.94	9 (50.0)	14 (63.6)	0.753^†^	0.385
LCX	69 (26.0)	96 (31.8)	2.262^†^	0.133	3 (16.7)	5 (22.7)	−	0.632
RCA	124 (46.8)	169 (56.0)	4.751^†^	0.029	11 (61.1)	11 (50.0)	0.494^†^	0.482
Multivessel disease Procedural issues, *n* (%)	77 (29.1)	124 (41.1)	8.887^†^	0.003	8 (44.4)	12 (54.5)	0.404^†^	0.525
**Procedural issues, *n* (%)**								
Radial access	254 (95.8)	276 (91.4)	4.599^†^	0.032	17 (94.4)	22 (100.0)	−	0.45
Stent use	236 (89.1)	281 (93.0)	2.794^†^	0.095	17 (94.4)	22 (100.0)	−	0.45
Direct stenting	100 (37.7)	86 (28.5)	5.489^†^	0.019	11 (61.1)	16 (72.7)	0.609^†^	0.435
Thrombus aspiration	34 (12.8)	22 (7.3)	4.876^†^	0.027	2 (11.1)	0 (0.0)	−	0.196
IABP use	16 (6.0)	22 (7.3)	0.351^†^	0.554	2 (11.1)	10 (45.5)	5.560^†^	0.018
ECMO use	2 (0.8)	1 (0.3)	−	0.602	0 (0.0)	0 (0.0)	−	−
Procedural success	259 (97.7)	299 (99.0)	−	0.225	18 (100.0)	22 (100.0)	−	−
Complications	4 (1.5)	4 (1.3)	−	1	1 (5.6)	0 (0.0)	−	0.45
**Initial TIMI flow grade (pre-PCI), *n* (%)**			0.774^†^	0.379			0.609^†^	0.435
TIMI flow grade 0–1	223 (84.2)	262 (86.8)			11 (61.1)	16 (72.7)		
TIMI flow grade 2–3	42 (15.8)	40 (13.2)			7 (38.9)	6 (27.3)		
**Final TIMI flow grade (post-PCI), n (%)**			−	1.000			−	−
TIMI flow grade 0–1	2 (0.8)	2 (0.7)			0 (0.0)	0 (0.0)		
TIMI flow grade 2–3	263 (99.2)	300 (99.3)			18 (100.0)	22 (100.0)		

*Data are expressed as mean ± SD, as percentages, or as median (Q1, Q3). ^†^χ2 value; ^‡^Z value. —: Data not available (Fisher exact test). COVID-19, coronavirus disease 2019; PCI, percutaneous coronary intervention; PPCI, primary PCI; LM, left main; LAD, left anterior descending; LCX, left circumflex; RCA, right coronary artery; IABP, intra-aortic balloon pump; ECMO, extracorporeal membrane oxygenation; TIMI, thrombolysis in myocardial infarction.*

### Clinical Outcomes

The in-hospital outcomes are shown in Table 3. No significant difference was observed in the median hospital length of stay between these two groups. There was no significant difference in in-hospital mortality between these two groups (2.4 vs. 3.4%, *P* = 0.490). One non-fatal myocardial infarction occurred in pre-COVID-19 era group. Two patients in the COVID-19 era group and one in the pre-COVID-19 era group experienced non-fatal stroke in hospital in COVID-19 era group. The rate of in-hospital heart failure decreased from 8.0 to 4.9% during the outbreak period. The rate of target vessel revascularization increased slightly from 0.9 to 2.4% during the outbreak period. Finally, the cumulative MACEs were similar between two groups (9.8 vs. 10.8%, *p* = 0.682). The adjusted odds of in-hospital death and MACEs are shown in Table 4. Following adjustment for covariates, no significant differences were found for in-hospital death (odds ratio [OR] 1.180, 95% confidence interval [CI] 0.181–7.679, *P* = 0.862) or MACEs (OR 1.390, 95%CI 0.612–3.161, *P* = 0.431).

**TABLE 3:** Clinical outcomes data.

	COVID-19 era group (*n* = 286)	Pre-COVID-19 era (*n* = 324)	Statistic	*P* Value
Length of stay, *d*	8 (6–10)	8 (6–11)	−0.988^‡^	0.323
In-hospital death, *n* (%)	7 (2.4)	11 (3.4)	0.476^†^	0.490
Non-fatal MI, *n* (%)	0 (0.0)	1 (0.3)	−	1.000
Non-fatal stroke, *n* (%)	2 (0.7)	1 (0.3)	−	0.602
Congestive heart failure, *n* (%)	13 (4.5)	24 (7.4)	2.184^†^	0.139
Target vessel revascularization, *n* (%)	7 (2.4)	3 (0.9)	−	0.202
Cumulative MACEs, *n* (%)	29 (10.1)	40 (12.3)	0.737^†^	0.391

*Data are expressed as mean ± SD, as percentages, or as median (Q_1_, Q_3_). ^†^χ^2^ value; ^‡^Z value. —: Data not available (Fisher exact test). COVID-19, coronavirus disease 2019; MI, myocardial infarction; MACEs, major adverse cardiovascular events.*

**TABLE 4:** Multivariate logistic regression analysis.

	Comparison of COVID-19 era group versus Pre-COVID-19 era group	
	Adjusted OR (95% CI)*	*P* value
In-hospital death	3.935 (0.511, 30.310)	0.188
MACEs	1.074 (0.416, 2.770)	0.883

COVID-19, coronavirus disease 2019; MACEs, major adverse cardiovascular events. *Adjusted for age, sex, hypertension, hypercholesterolemia, diabetes mellitus, smokers, previous myocardial infarction, previous percutaneous coronary intervention, previous coronary artery bypass graft, aspirin, P2Y12 receptor antagonist, glycoprotein IIb/IIIa inhibitor use, β-blockers, statins, angiotensin converting enzyme inhibitors/Angiotensin II receptor blockers, symptom-to-hospital time, door-to-balloon time, radial access, multivessel disease, vessel of intervention, flow, intra-aortic balloon pump, extracorporeal membrane oxygenation.

## Discussion

In response to the COVID-19 pandemic, many countries have implemented strict infection containment measures such as “lockdown” and encouraged a “stay-at-home” lifestyle, to reduce the spread of the pandemic (8, 9, 15). Moreover, the routine hospital services including cardiac catheterization have been restructured in order to increase hospital capacity for COVID-19 patients and prevent cross-infection. These strict restriction measures would inevitably have a profound impact on routine medical care, in particular, acute cardiovascular disease management.

In the present study, we conducted a retrospective analysis in 610 STEMI patients receiving acute reperfusion treatment including PPCI, pharmaco-invasive strategy and systematic thrombolysis and compared the in-hospital clinical outcomes of patients presenting during the COVID-19 pandemic vs. pre-COVID-19 era. First, we demonstrated a 10.5% drop in STEMI volumes, a 12.7% decline in STEMI-related PCI procedures and a significantly higher cTnI level on admission during the COVID-19 outbreak. Second, in terms of time delay, the pandemic of COVID-19 incurred no additional time delay whether in the PPCI subgroup or pharmaco-invasive strategy subgroup. Finally, there were no differences in clinical outcomes including in-hospital mortality and MACEs before and after lockdown.

Previous studies have reported a common decrease in STEMI admissions while the degree of decline varied considerably among countries affected by the COVID-19 pandemic. During the early phase of the COVID-19 pandemic, Xiang et al. (15) reported a 26.3% reduction in STEMI patients’ access to care in non-Hubei provinces in China based on the Chest Pain Center database. A similar reduction of 23% in admissions for STEMI was reported in England (9). Scholz et al. (16) reported a mild decrease in the absolute number of STEM patients treated in systems of STEMI care in Germany (12.6%). Our results supported this finding but presented a relatively milder decrease in STEMI volumes (10.5%). In contrast, reports from other countries (e.g., Singapore, France, and Denmark) report no appreciable decrease and even a modest increase in STEMI volumes (17–19). The largely discrepant reports of STEMI hospitalization across countries could be partly explained by disparities in healthcare organizations.

Multiple factors might contribute to this decline in admissions of patients with STEMI during the COVID-19 pandemic. One possibility is that the case of misdiagnosis increased because of complex cardiovascular manifestations under the circumstance of COVID-19. It is challenging to differentiate STEMI patients from COVID-19 patients, who might simulate a STEMI manifestation and present with cardiac troponin elevation and/or ST changes (20). Therefore, a proportion of critical STEMI with dyspnea and pulmonary edema could be mistaken with the coronavirus features and managed as a COVID-19 case from the outset. The fear of medical system might be another important factor. The soaring confirmed infections, no effective therapeutic drugs, no vaccines and lack of personal protective equipment may have created an atmosphere of fear. The symptomatic patients might avoid seeking acute medical care for fear of getting in contact with COVID-19 patients (21).

Additionally, our finding showed a decline of 12.7% in STEMI-related PCI procedures, which supports the decline in PCI procedures for STEMI reported in other studies, but we add some additional value to such observations by describing clinical and procedural characteristics and outcomes after the COVID-19 lockdown using last year as a reference. A preliminary analysis from multiple United States centers showed during the early phase of the COVID-19 pandemic, an estimated 38% reduction in cardiac catheterization laboratories activations for STEMI care (22). Another survey of 73 centers in Spain reported a 40% reduction in procedures performed in the STEMI settings (23). Using the British Cardiovascular Intervention Society database, Kwok et al. (24) reported a 43% reduction in all STEMI-related PCI procedures in England in the month after the lockdown. In the present study, we observed a slight decline in STEMI-related PCI procedures. The different degrees of decline in nations and regions indicated the huge differences in terms of local healthcare resources, the pandemic density of the COVID-19 outbreak and changes of the pandemic over time. In China, since Hubei province started lockdown on January 23, 2020, Hunan province had activated Level one major public health emergency response on the same day. With the joint efforts of the government and people, the epidemic was quickly controlled. Subsequently, the government degraded the major public health emergency response to Level two on March 10 due to a sustained decrease in the number of new cases. The regional medical system had the capacity to continue to provide emergency STEMI care according to current clinical practice guidelines.

Undoubtedly, the COVID-19 pandemic is a major burden on the time-dependent emergency healthcare networks and is imposing a change on STEMI care especially in region heavy involvement in the epidemic. An important issue merit consideration is how changes in patients’ health-seeking behavior, health service delivery and government strategies to restrict virus spread impact clinical characteristics and outcomes of the patients (24). Our study showed patients admitted during the COVID-19 pandemic were more likely to have a history of previous PCI with a significant increase in the baseline cTnI level compared to a similar time frame last year. Similar observations have also been reported from England and Germany (21, 25). The fear of getting infected within the hospitals and government calls to stay at home and seek medical care only in case of an emergency may lead to patients’ delay seeking a doctor, and aggravation of their symptoms (21, 24). As a consequence, a substantial reduction in admissions for STEMI and an increase in the number of out-of-hospital cardiac arrests were observed (26). In the present study, we found relatively fewer patients receiving PCI during the COVID-19 pandemic and no overall increase in in-hospital mortality and MACEs among patients admitted for STEMI. Despite this fact, caution must be exercised in interpreting the results. On the one hand, many patients had STEMI but receive no reperfusion therapy in hospital because of deaths out of hospital. On the other hand, it warrants much investigation to assess whether the long-term clinical outcome was not different before and after the COVID-19 pandemic outbreak.

In present study, there was no difference between two groups in terms of key time interval and short term in-hospital outcomes for STEM patients. This result was in line with studies in other regions of China. A single center report from Beijing by Guan et al. (27) showed door to balloon time, operation time and the incidence of MACEs were similar pre and during COVID-19 pandemic. Similar results were found in Shenzhen, a metropolitan city in southern of China (28). Therefore, above results indicated that safety measures to prevent nosocomial COVID-19 infection did not compromise the in-hospital outcomes as compared with PCI under normal condition. The regional collaborative STEMI treatment network established in China worked well and ensured timely acute cardiac care even in the context of the COVID-19 pandemic.

Our data demonstrated that a better public communication approach should be adopted to reassure patients in critical conditions to obtain timely medical contact. Public health, political, and physician leaders in China have taken aggressive measures to encourage patients with heart attack symptoms to seek medical care. Social media including WeChat, Weibo, Tik Tok, and so on was applied as a tool for grassroots health promotion initiatives during the COVID-19 pandemic. Based on social media platforms, healthcare professionals reeducated the general population to recognize and act on heart attack signs and symptoms and call an ambulance immediately. Furthermore, it’s necessary to stress that the national healthcare system still had the capacity to provide prompt and effective care in a manner that was safe for both patients and healthcare workers. Meanwhile, hospitals had to take appropriate precautions to protect patients and healthcare workers from COVID-19 infection.

## Study Limitations

Our study has several limitations. First, although patients affected by COVID-19 were excluded from the final analysis, we cannot definitively exclude the possibility that patients in the COVID-19 era may have COVID-19 infection because it’s hard to make an absolutely accurate diagnosis in the early phase of the pandemic. However, we believe this possibility was very small because all enrolled patients were lack of the epidemiological history and clinical manifestations. Second, we assessed only in-hospital outcomes, as data on post-discharge follow-up are currently not available. Third, the onset of symptoms was a subjective parameter and might not be precisely recorded. Finally, self-report of in-hospital outcomes generally along with early discharge may have resulted in under-reporting of adverse outcomes.

## Future Directions

Every effort should be made to educate the public to recognize symptoms of life-threatening cardiac conditions and seek appropriate care in a timely fashion. Health authorities should implement strategies to further optimize the STEMI care system in response to emerging infectious diseases like COVID-19.

## Conclusion

The COVID-19 pandemic outbreak led to a decline in the number of admitted STEMI cases as well as STEMI-related PCI procedures in Hunan province, China. The key quality indicators of reperfusion treatment including median time from symptom onset to FMC, symptom onset to door, door-to-balloon, symptom onset to balloon, and symptom onset to thrombolysis, were not significantly affected during the pandemic outbreak. Restructuring health services during the COVID-19 pandemic has not significantly adversely influenced the in-hospital outcomes.

## Data Availability Statement

The original contributions presented in the study are included in the article/supplementary material, further inquiries can be directed to the corresponding authors.

## Ethics Statement

The studies involving human participants were reviewed and approved by Ethics Committee of The Second Xiangya Hospital, Central South University. The patients/participants provided their written informed consent to participate in this study.

## Author Contributions

LT and Z-JW participated in the design of the study, collected clinical data, performed statistical analysis, and drafted the manuscript. X-QH, Z-FF, Z-FZ, J-PZ, L-PJ, FO, C-HL, and G-FZ participated in the treatment for the patients and collected clinical data. Y-HG and S-HZ participated in the design of the study, revised the final version of the manuscript, and supervised the study. All authors participated in the research and reviewed the final version of the manuscript.

## Conflict of Interest

The authors declare that the research was conducted in the absence of any commercial or financial relationships that could be construed as a potential conflict of interest.

## Publisher’s Note

All claims expressed in this article are solely those of the authors and do not necessarily represent those of their affiliated organizations, or those of the publisher, the editors and the reviewers. Any product that may be evaluated in this article, or claim that may be made by its manufacturer, is not guaranteed or endorsed by the publisher.
